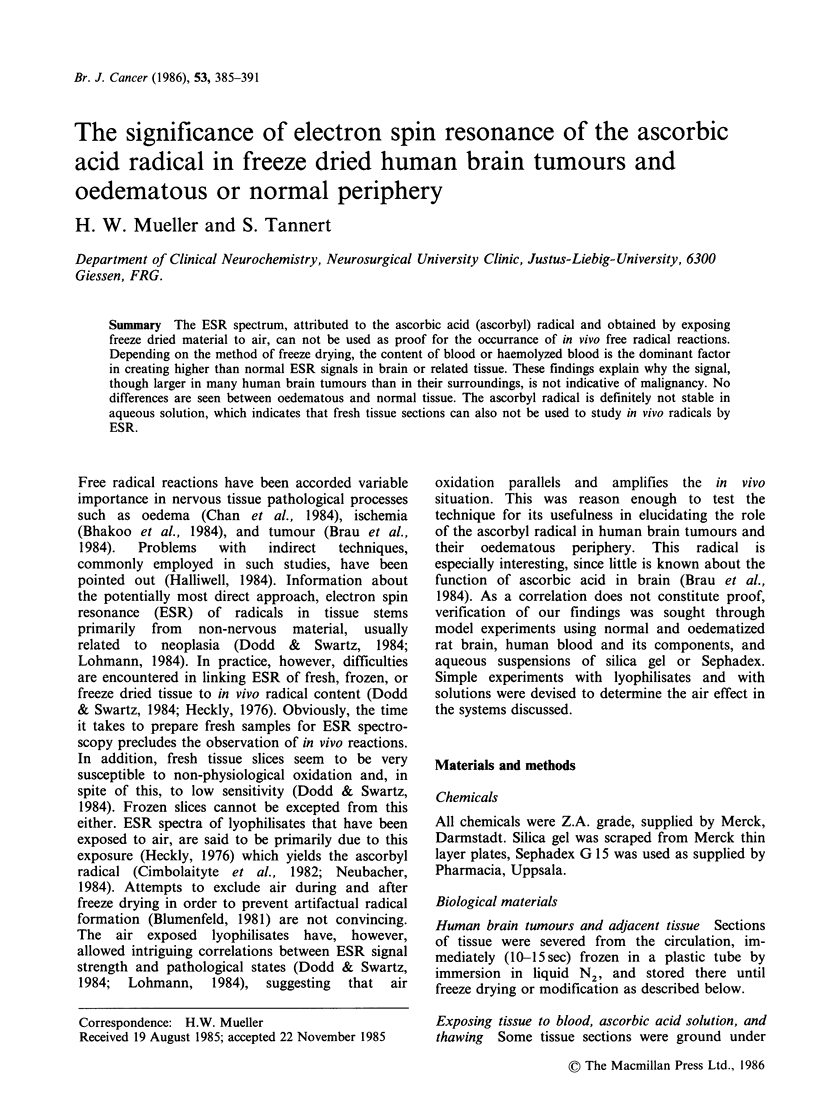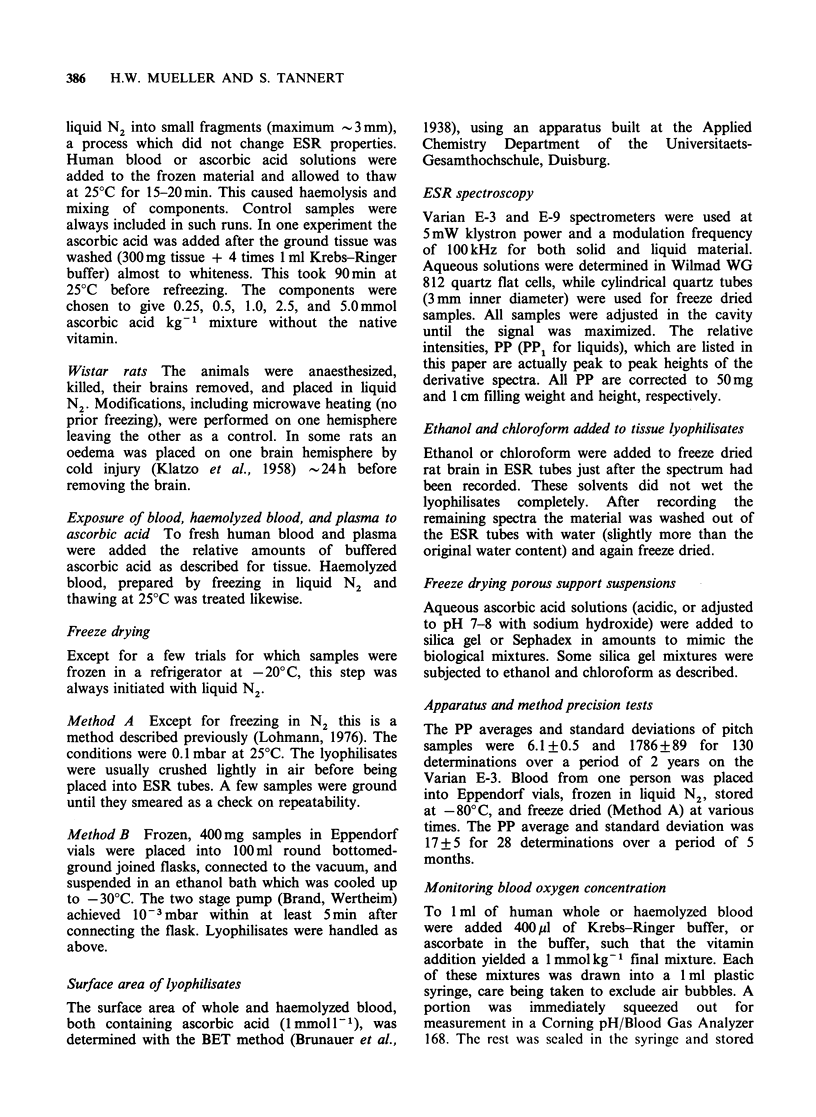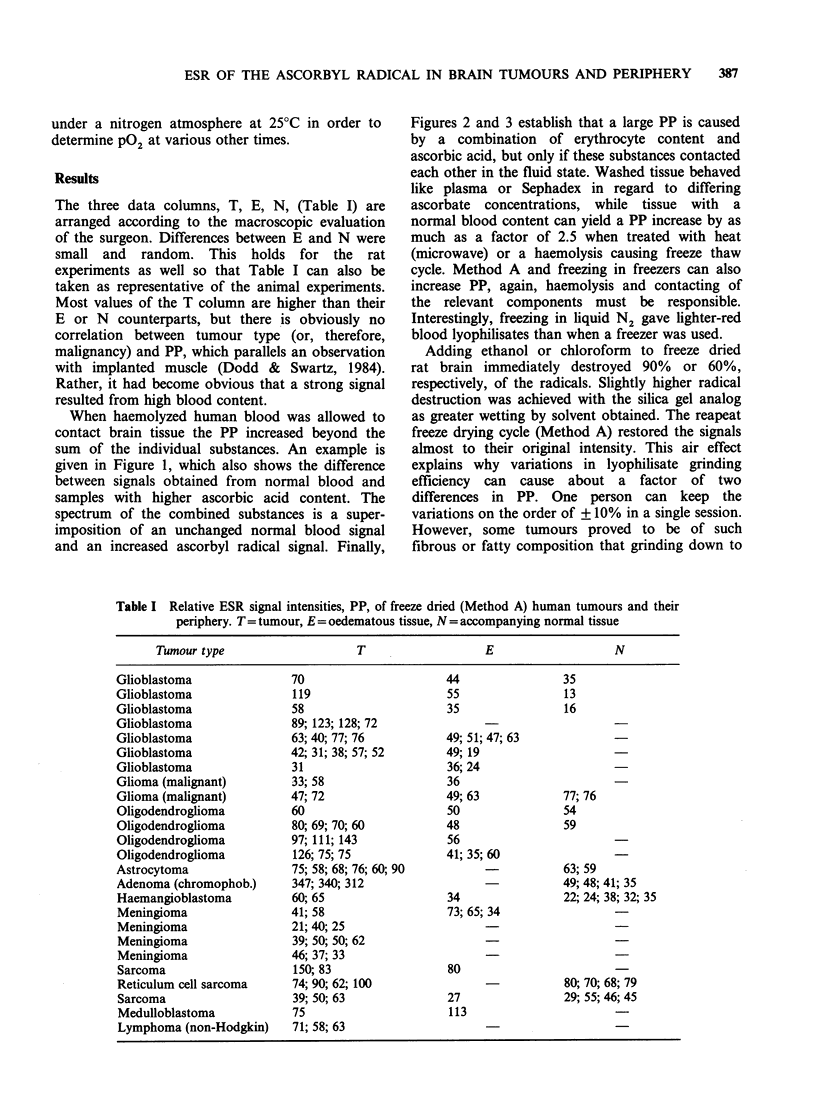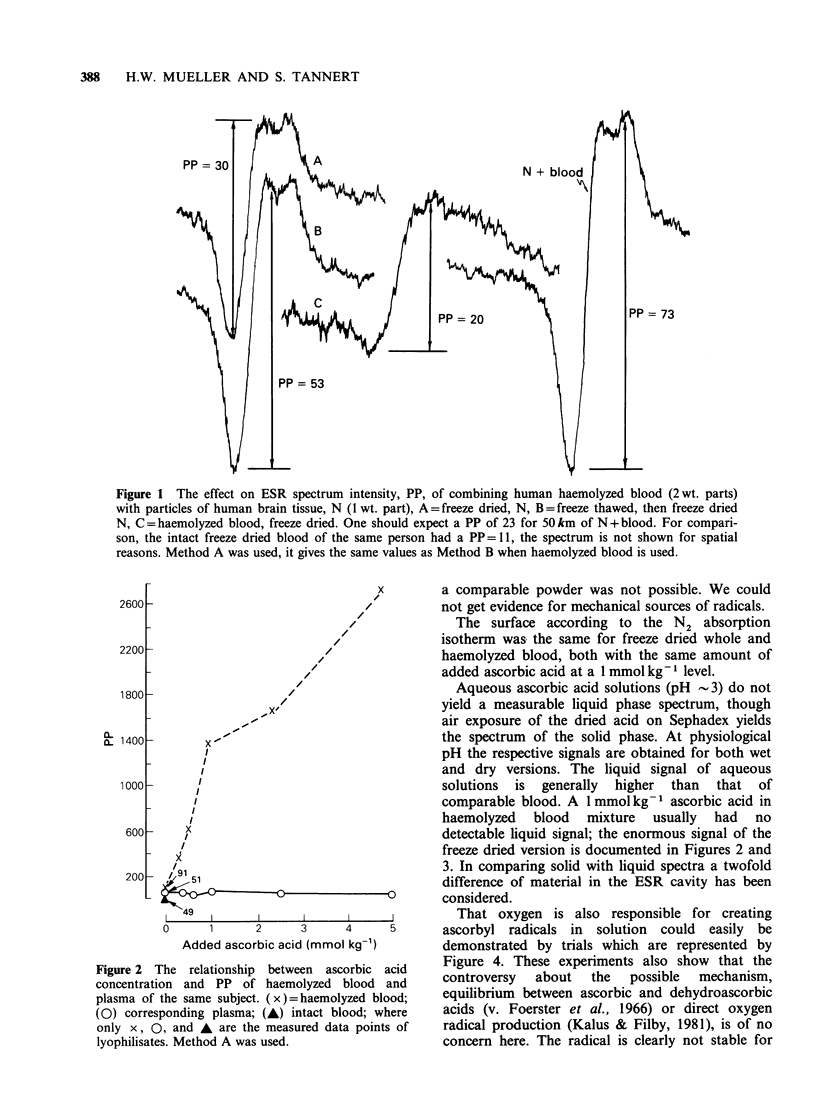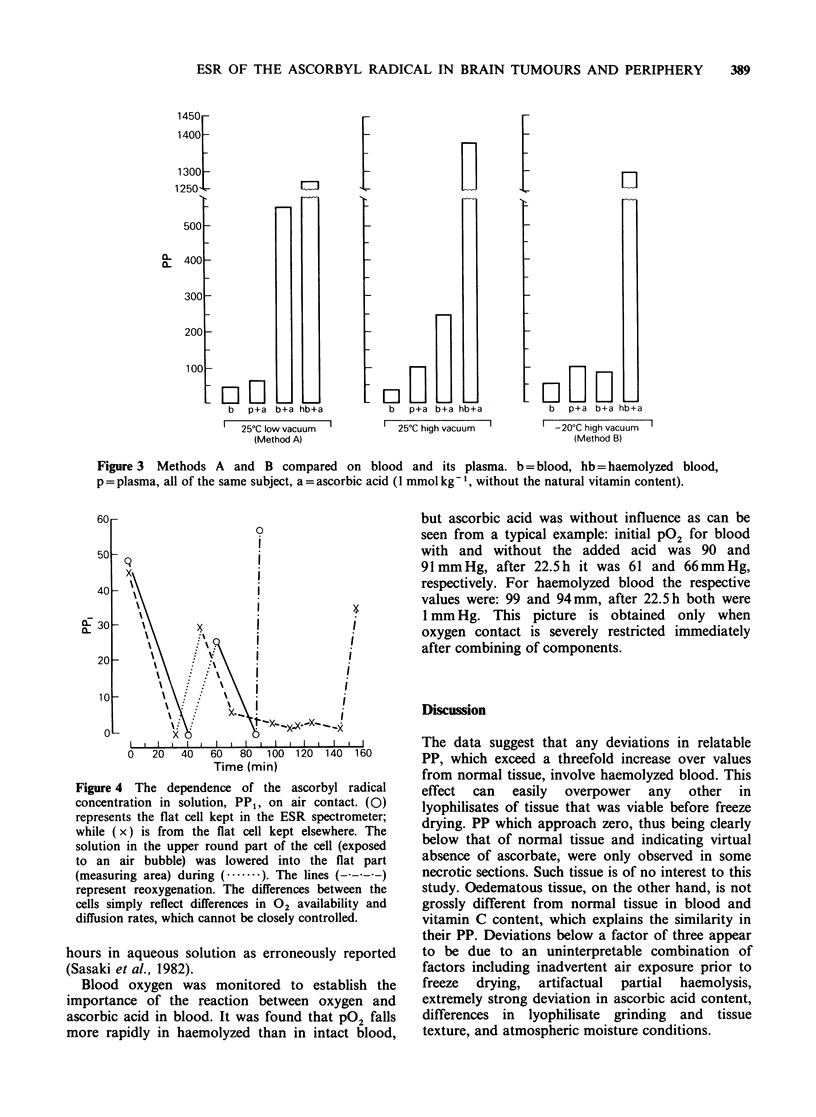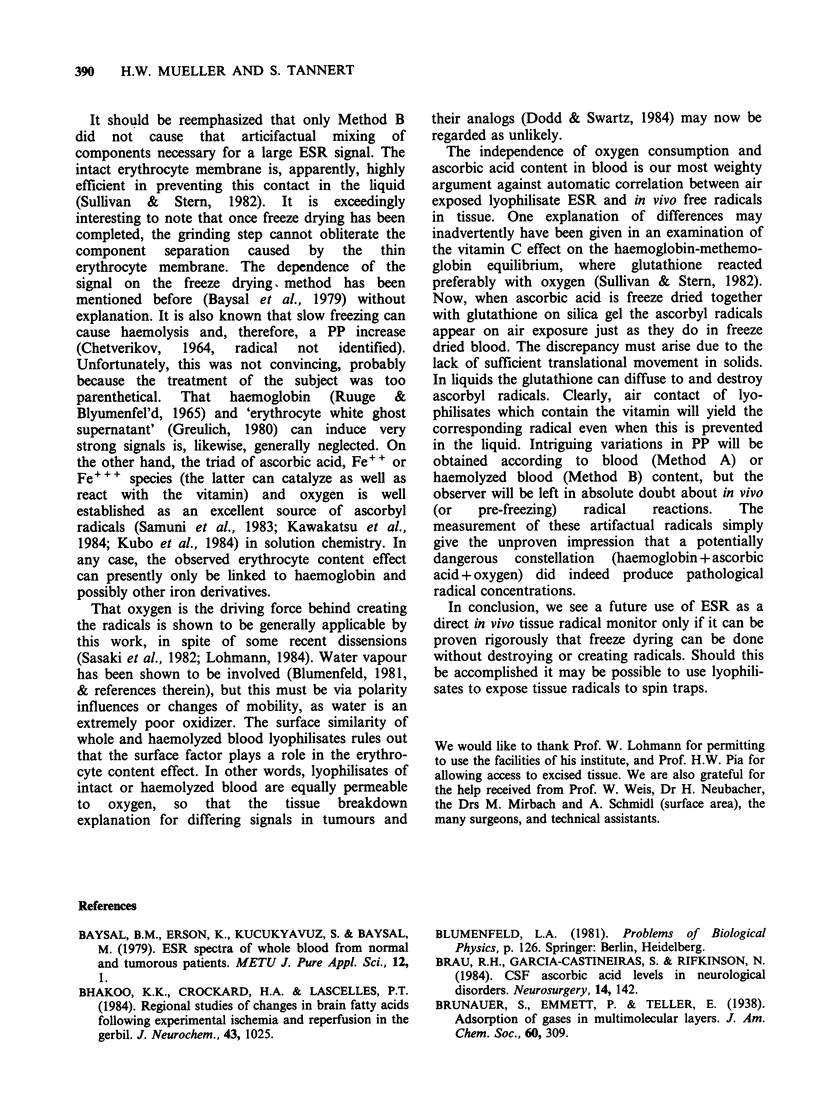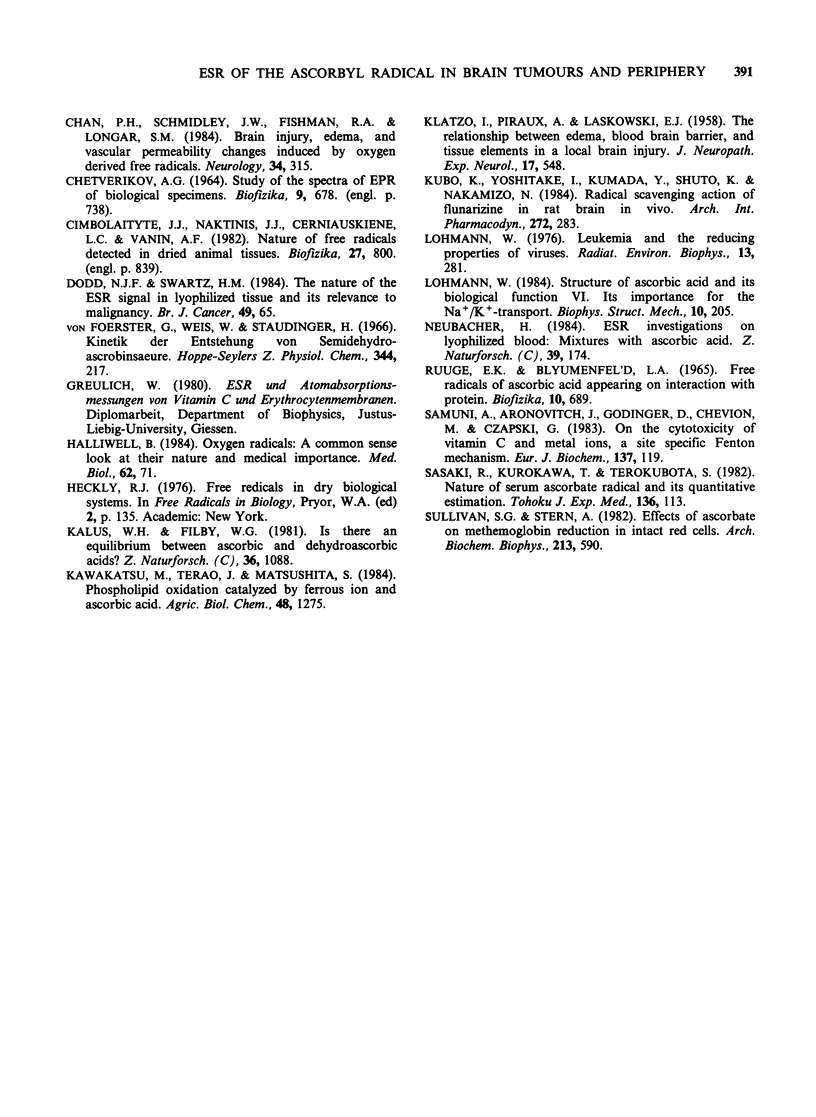# The significance of electron spin resonance of the ascorbic acid radical in freeze dried human brain tumours and oedematous or normal periphery.

**DOI:** 10.1038/bjc.1986.63

**Published:** 1986-03

**Authors:** H. W. Mueller, S. Tannert

## Abstract

The ESR spectrum, attributed to the ascorbic acid (ascorbyl) radical and obtained by exposing freeze dried material to air, can not be used as proof for the occurrence of in vivo free radical reactions. Depending on the method of freeze drying, the content of blood or hemolyzed blood is the dominant factor in creating higher than normal ESR signals in brain or related tissue. These findings explain why the signal, though larger in many human brain tumours than in their surroundings, is not indicative of malignancy. No differences are seen between oedematous and normal tissue. The ascorbyl radical is definitely not stable in aqueous solution, which indicates that fresh tissue sections can also not be used to study in vivo radicals by ESR.


*Br. J. Cancer* (1986), **53**, 385–391

### The significance of electron spin resonance of the ascorbic acid radical in freeze dried human brain tumours and oedematous or normal periphery

H. W. Mueller and S. Tannert

*Department of Clinical Neurochemistry, Neurosurgical University Clinic, Justus-Liebig-University, 6300 Giessen, FRG.*

**Summary** The ESR spectrum, attributed to the ascorbic acid (ascorbyl) radical and obtained by exposing freeze dried material to air, can not be used as proof for the occurrance of *in vivo* free radical reactions. Depending on the method of freeze drying, the content of blood or haemolyzed blood is the dominant factor in creating higher than normal ESR signals in brain or related tissue. These findings explain why the signal, though larger in many human brain tumours than in their surroundings, is not indicative of malignancy. No differences are seen between oedematous and normal tissue. The ascorbyl radical is definitely not stable in aqueous solution, which indicates that fresh tissue sections can also not be used to study *in vivo* radicals by ESR.

Free radical reactions have been accorded variable importance in nervous tissue pathological processes such as oedema (Chan *et al.,* 1984), ischemia (Bhakoo *et al.,* 1984), and tumour (Brau *et al.,* 1984). Problems with indirect techniques, commonly employed in such studies, have been pointed out (Halliwell, 1984). Information about the potentially most direct approach, electron spin resonance (ESR) of radicals in tissue stems primarily from non-nervous material, usually related to neoplasia (Dodd & Swartz, 1984; Lohmann, 1984). In practice, however, difficulties are encountered in linking ESR of fresh, frozen, or freeze dried tissue to *in vivo* radical content (Dodd & Swartz, 1984; Heckly, 1976). Obviously, the time it takes to prepare fresh samples for ESR spectroscopy precludes the observation of *in vivo* reactions. In addition, fresh tissue slices seem to be very susceptible to non-physiological oxidation and, in spite of this, to low sensitivity (Dodd & Swartz, 1984). Frozen slices cannot be excepted from this either. ESR spectra of lyophilisates that have been exposed to air, are said to be primarily due to this exposure (Heckly, 1976) which yields the ascorbyl radical (Cimbolaityte *et al.,* 1982; Neubacher, 1984). Attempts to exclude air during and after freeze drying in order to prevent artifactual radical formation (Blumenfeld, 1981) are not convincing. The air exposed lyophilisates have, however, allowed intriguing correlations between ESR signal strength and pathological states (Dodd & Swartz, 1984; Lohmann, 1984), suggesting that air oxidation parallels and amplifies the *in vivo* situation. This was reason enough to test the technique for its usefulness in elucidating the role of the ascorbyl radical in human brain tumours and their oedematous periphery. This radical is especially interesting, since little is known about the function of ascorbic acid in brain (Brau *et al.,* 1984). As a correlation does not constitute proof, verification of our findings was sought through model experiments using normal and oedematized rat brain, human blood and its components, and aqueous suspensions of silica gel or Sephadex. Simple experiments with lyophilisates and with solutions were devised to determine the air effect in the systems discussed.

Correspondence: H.W. Mueller
Received 19 August 1985; accepted 22 November 1985

### Materials and methods

### Chemicals

All chemicals were Z.A. grade, supplied by Merck, Darmstadt. Silica gel was scraped from Merck thin layer plates, Sephadex G 15 was used as supplied by Pharmacia, Uppsala.

### Biological materials

*Human brain tumours and adjacent tissue* Sections of tissue were severed from the circulation, immediately (10–15 sec) frozen in a plastic tube by immersion in liquid $N_2$, and stored there until freeze drying or modification as described below.

*Exposing tissue to blood, ascorbic acid solution, and thawing* Some tissue sections were ground under

© The Macmillan Press Ltd., 1986

liquid $N_2$ into small fragments (maximum ~3 mm), a process which did not change ESR properties. Human blood or ascorbic acid solutions were added to the frozen material and allowed to thaw at 25°C for 15–20 min. This caused haemolysis and mixing of components. Control samples were always included in such runs. In one experiment the ascorbic acid was added after the ground tissue was washed (300 mg tissue + 4 times 1 ml Krebs–Ringer buffer) almost to whiteness. This took 90 min at 25°C before refreezing. The components were chosen to give 0.25, 0.5, 1.0, 2.5, and 5.0 mmol ascorbic acid $kg^{-1}$ mixture without the native vitamin.

*Wistar rats* The animals were anaesthesized, killed, their brains removed, and placed in liquid $N_2$. Modifications, including microwave heating (no prior freezing), were performed on one hemisphere leaving the other as a control. In some rats an oedema was placed on one brain hemisphere by cold injury (Klatzo *et al.*, 1958) ~24 h before removing the brain.

*Exposure of blood, haemolyzed blood, and plasma to ascorbic acid* To fresh human blood and plasma were added the relative amounts of buffered ascorbic acid as described for tissue. Haemolyzed blood, prepared by freezing in liquid $N_2$ and thawing at 25°C was treated likewise.

*Freeze drying*

Except for a few trials for which samples were frozen in a refrigerator at −20°C, this step was always initiated with liquid $N_2$.

*Method A* Except for freezing in $N_2$ this is a method described previously (Lohmann, 1976). The conditions were 0.1 mbar at 25°C. The lyophilisates were usually crushed lightly in air before being placed into ESR tubes. A few samples were ground until they smeared as a check on repeatability.

*Method B* Frozen, 400 mg samples in Eppendorf vials were placed into 100 ml round bottomed-ground joined flasks, connected to the vacuum, and suspended in an ethanol bath which was cooled up to −30°C. The two stage pump (Brand, Wertheim) achieved $10^{-3}$ mbar within at least 5 min after connecting the flask. Lyophilisates were handled as above.

*Surface area of lyophilisates*

The surface area of whole and haemolyzed blood, both containing ascorbic acid (1 mmol $l^{-1}$), was determined with the BET method (Brunauer *et al.*, 1938), using an apparatus built at the Applied Chemistry Department of the Universitaets-Gesamthochschule, Duisburg.

*ESR spectroscopy*

Varian E-3 and E-9 spectrometers were used at 5 mW klystron power and a modulation frequency of 100 kHz for both solid and liquid material. Aqueous solutions were determined in Wilmad WG 812 quartz flat cells, while cylindrical quartz tubes (3 mm inner diameter) were used for freeze dried samples. All samples were adjusted in the cavity until the signal was maximized. The relative intensities, PP ($PP_1$ for liquids), which are listed in this paper are actually peak to peak heights of the derivative spectra. All PP are corrected to 50 mg and 1 cm filling weight and height, respectively.

*Ethanol and chloroform added to tissue lyophilisates*

Ethanol or chloroform were added to freeze dried rat brain in ESR tubes just after the spectrum had been recorded. These solvents did not wet the lyophilisates completely. After recording the remaining spectra the material was washed out of the ESR tubes with water (slightly more than the original water content) and again freeze dried.

*Freeze drying porous support suspensions*

Aqueous ascorbic acid solutions (acidic, or adjusted to pH 7–8 with sodium hydroxide) were added to silica gel or Sephadex in amounts to mimic the biological mixtures. Some silica gel mixtures were subjected to ethanol and chloroform as described.

*Apparatus and method precision tests*

The PP averages and standard deviations of pitch samples were $6.1 \pm 0.5$ and $1786 \pm 89$ for 130 determinations over a period of 2 years on the Varian E-3. Blood from one person was placed into Eppendorf vials, frozen in liquid $N_2$, stored at −80°C, and freeze dried (Method A) at various times. The PP average and standard deviation was $17 \pm 5$ for 28 determinations over a period of 5 months.

*Monitoring blood oxygen concentration*

To 1 ml of human whole or haemolyzed blood were added 400 $\mu$l of Krebs–Ringer buffer, or ascorbate in the buffer, such that the vitamin addition yielded a 1 mmol $kg^{-1}$ final mixture. Each of these mixtures was drawn into a 1 ml plastic syringe, care being taken to exclude air bubbles. A portion was immediately squeezed out for measurement in a Corning pH/Blood Gas Analyzer 168. The rest was sealed in the syringe and stored under a nitrogen atmosphere at 25°C in order to determine $pO_2$ at various other times.

### Results

The three data columns, T, E, N, (Table I) are arranged according to the macroscopic evaluation of the surgeon. Differences between E and N were small and random. This holds for the rat experiments as well so that Table I can also be taken as representative of the animal experiments. Most values of the T column are higher than their E or N counterparts, but there is obviously no correlation between tumour type (or, therefore, malignancy) and PP, which parallels an observation with implanted muscle (Dodd & Swartz, 1984). Rather, it had become obvious that a strong signal resulted from high blood content.

When haemolyzed human blood was allowed to contact brain tissue the PP increased beyond the sum of the individual substances. An example is given in Figure 1, which also shows the difference between signals obtained from normal blood and samples with higher ascorbic acid content. The spectrum of the combined substances is a superimposition of an unchanged normal blood signal and an increased ascorbyl radical signal. Finally, Figures 2 and 3 establish that a large PP is caused by a combination of erythrocyte content and ascorbic acid, but only if these substances contacted each other in the fluid state. Washed tissue behaved like plasma or Sephadex in regard to differing ascorbate concentrations, while tissue with a normal blood content can yield a PP increase by as much as a factor of 2.5 when treated with heat (microwave) or a haemolysis causing freeze thaw cycle. Method A and freezing in freezers can also increase PP, again, haemolysis and contacting of the relevant components must be responsible. Interestingly, freezing in liquid $N_2$ gave lighter-red blood lyophilisates than when a freezer was used.

Adding ethanol or chloroform to freeze dried rat brain immediately destroyed 90% or 60%, respectively, of the radicals. Slightly higher radical destruction was achieved with the silica gel analog as greater wetting by solvent obtained. The reapeat freeze drying cycle (Method A) restored the signals almost to their original intensity. This air effect explains why variations in lyophilisate grinding efficiency can cause about a factor of two differences in PP. One person can keep the variations on the order of $\pm 10\%$ in a single session. However, some tumours proved to be of such fibrous or fatty composition that grinding down to

**Table I** Relative ESR signal intensities, PP, of freeze dried (Method A) human tumours and their periphery. $T$ = tumour, $E$ = oedematous tissue, $N$ = accompanying normal tissue

| *Tumour type* | *T* | *E* | *N* |
|---|---|---|---|
| Glioblastoma | 70 | 44 | 35 |
| Glioblastoma | 119 | 55 | 13 |
| Glioblastoma | 58 | 35 | 16 |
| Glioblastoma | 89; 123; 128; 72 | — | — |
| Glioblastoma | 63; 40; 77; 76 | 49; 51; 47; 63 | — |
| Glioblastoma | 42; 31; 38; 57; 52 | 49; 19 | — |
| Glioblastoma | 31 | 36; 24 | — |
| Glioma (malignant) | 33; 58 | 36 | — |
| Glioma (malignant) | 47; 72 | 49; 63 | 77; 76 |
| Oligodendroglioma | 60 | 50 | 54 |
| Oligodendroglioma | 80; 69; 70; 60 | 48 | 59 |
| Oligodendroglioma | 97; 111; 143 | 56 | — |
| Oligodendroglioma | 126; 75; 75 | 41; 35; 60 | — |
| Astrocytoma | 75; 58; 68; 76; 60; 90 | — | 63; 59 |
| Adenoma (chromophob.) | 347; 340; 312 | — | 49; 48; 41; 35 |
| Haemangioblastoma | 60; 65 | 34 | 22; 24; 38; 32; 35 |
| Meningioma | 41; 58 | 73; 65; 34 | — |
| Meningioma | 21; 40; 25 | — | — |
| Meningioma | 39; 50; 50; 62 | — | — |
| Meningioma | 46; 37; 33 | — | — |
| Sarcoma | 150; 83 | 80 | — |
| Reticulum cell sarcoma | 74; 90; 62; 100 | — | 80; 70; 68; 79 |
| Sarcoma | 39; 50; 63 | 27 | 29; 55; 46; 45 |
| Medulloblastoma | 75 | 113 | — |
| Lymphoma (non-Hodgkin) | 71; 58; 63 | — | — |

**Figure 1** The effect on ESR spectrum intensity, PP, of combining human haemolyzed blood (2 wt. parts) with particles of human brain tissue, N (1 wt. part), A = freeze dried, N, B = freeze thawed, then freeze dried N, C = haemolyzed blood, freeze dried. One should expect a PP of 23 for 50 *k*m of N + blood. For comparison, the intact freeze dried blood of the same person had a PP = 11, the spectrum is not shown for spatial reasons. Method A was used, it gives the same values as Method B when haemolyzed blood is used.

**Figure 2** The relationship between ascorbic acid concentration and PP of haemolyzed blood and plasma of the same subject. (×) = haemolyzed blood; (○) corresponding plasma; (▲) intact blood; where only ×, ○, and ▲ are the measured data points of lyophilisates. Method A was used.

a comparable powder was not possible. We could not get evidence for mechanical sources of radicals.

The surface according to the $N_2$ absorption isotherm was the same for freeze dried whole and haemolyzed blood, both with the same amount of added ascorbic acid at a 1 mmol kg$^{-1}$ level.

Aqueous ascorbic acid solutions (pH ~3) do not yield a measurable liquid phase spectrum, though air exposure of the dried acid on Sephadex yields the spectrum of the solid phase. At physiological pH the respective signals are obtained for both wet and dry versions. The liquid signal of aqueous solutions is generally higher than that of comparable blood. A 1 mmol kg$^{-1}$ ascorbic acid in haemolyzed blood mixture usually had no detectable liquid signal; the enormous signal of the freeze dried version is documented in Figures 2 and 3. In comparing solid with liquid spectra a twofold difference of material in the ESR cavity has been considered.

That oxygen is also responsible for creating ascorbyl radicals in solution could easily be demonstrated by trials which are represented by Figure 4. These experiments also show that the controversy about the possible mechanism, equilibrium between ascorbic and dehydroascorbic acids (v. Foerster *et al.*, 1966) or direct oxygen radical production (Kalus & Filby, 1981), is of no concern here. The radical is clearly not stable for

**Figure 3** Methods A and B compared on blood and its plasma. b = blood, hb = haemolyzed blood, p = plasma, all of the same subject, a = ascorbic acid (1 mmol $kg^{-1}$, without the natural vitamin content).

**Figure 4** The dependence of the ascorbyl radical concentration in solution, $PP_l$, on air contact. (O) represents the flat cell kept in the ESR spectrometer; while (×) is from the flat cell kept elsewhere. The solution in the upper round part of the cell (exposed to an air bubble) was lowered into the flat part (measuring area) during (·······). The lines (–·–·–) represent reoxygenation. The differences between the cells simply reflect differences in $O_2$ availability and diffusion rates, which cannot be closely controlled.

hours in aqueous solution as erroneously reported (Sasaki *et al.*, 1982).

Blood oxygen was monitored to establish the importance of the reaction between oxygen and ascorbic acid in blood. It was found that $pO_2$ falls more rapidly in haemolyzed than in intact blood, but ascorbic acid was without influence as can be seen from a typical example: initial $pO_2$ for blood with and without the added acid was 90 and 91 mm Hg, after 22.5 h it was 61 and 66 mm Hg, respectively. For haemolyzed blood the respective values were: 99 and 94 mm, after 22.5 h both were 1 mm Hg. This picture is obtained only when oxygen contact is severely restricted immediately after combining of components.

### Discussion

The data suggest that any deviations in relatable PP, which exceed a threefold increase over values from normal tissue, involve haemolyzed blood. This effect can easily overpower any other in lyophilisates of tissue that was viable before freeze drying. PP which approach zero, thus being clearly below that of normal tissue and indicating virtual absence of ascorbate, were only observed in some necrotic sections. Such tissue is of no interest to this study. Oedematous tissue, on the other hand, is not grossly different from normal tissue in blood and vitamin C content, which explains the similarity in their PP. Deviations below a factor of three appear to be due to an uninterpretable combination of factors including inadvertent air exposure prior to freeze drying, artifactual partial haemolysis, extremely strong deviation in ascorbic acid content, differences in lyophilisate grinding and tissue texture, and atmospheric moisture conditions.

It should be reemphasized that only Method B did not cause that articifactual mixing of components necessary for a large ESR signal. The intact erythrocyte membrane is, apparently, highly efficient in preventing this contact in the liquid (Sullivan & Stern, 1982). It is exceedingly interesting to note that once freeze drying has been completed, the grinding step cannot obliterate the component separation caused by the thin erythrocyte membrane. The dependence of the signal on the freeze drying method has been mentioned before (Baysal *et al.*, 1979) without explanation. It is also known that slow freezing can cause haemolysis and, therefore, a PP increase (Chetverikov, 1964, radical not identified). Unfortunately, this was not convincing, probably because the treatment of the subject was too parenthetical. That haemoglobin (Ruuge & Blyumenfel'd, 1965) and 'erythrocyte white ghost supernatant' (Greulich, 1980) can induce very strong signals is, likewise, generally neglected. On the other hand, the triad of ascorbic acid, $Fe^{++}$ or $Fe^{+++}$ species (the latter can catalyze as well as react with the vitamin) and oxygen is well established as an excellent source of ascorbyl radicals (Samuni *et al.*, 1983; Kawakatsu *et al.*, 1984; Kubo *et al.*, 1984) in solution chemistry. In any case, the observed erythrocyte content effect can presently only be linked to haemoglobin and possibly other iron derivatives.

That oxygen is the driving force behind creating the radicals is shown to be generally applicable by this work, in spite of some recent dissensions (Sasaki *et al.*, 1982; Lohmann, 1984). Water vapour has been shown to be involved (Blumenfeld, 1981, & references therein), but this must be via polarity influences or changes of mobility, as water is an extremely poor oxidizer. The surface similarity of whole and haemolyzed blood lyophilisates rules out that the surface factor plays a role in the erythrocyte content effect. In other words, lyophilisates of intact or haemolyzed blood are equally permeable to oxygen, so that the tissue breakdown explanation for differing signals in tumours and their analogs (Dodd & Swartz, 1984) may now be regarded as unlikely.

The independence of oxygen consumption and ascorbic acid content in blood is our most weighty argument against automatic correlation between air exposed lyophilisate ESR and *in vivo* free radicals in tissue. One explanation of differences may inadvertently have been given in an examination of the vitamin C effect on the haemoglobin-methemoglobin equilibrium, where glutathione reacted preferably with oxygen (Sullivan & Stern, 1982). Now, when ascorbic acid is freeze dried together with glutathione on silica gel the ascorbyl radicals appear on air exposure just as they do in freeze dried blood. The discrepancy must arise due to the lack of sufficient translational movement in solids. In liquids the glutathione can diffuse to and destroy ascorbyl radicals. Clearly, air contact of lyophilisates which contain the vitamin will yield the corresponding radical even when this is prevented in the liquid. Intriguing variations in PP will be obtained according to blood (Method A) or haemolyzed blood (Method B) content, but the observer will be left in absolute doubt about *in vivo* (or pre-freezing) radical reactions. The measurement of these artifactual radicals simply give the unproven impression that a potentially dangerous constellation (haemoglobin + ascorbic acid + oxygen) did indeed produce pathological radical concentrations.

In conclusion, we see a future use of ESR as a direct *in vivo* tissue radical monitor only if it can be proven rigorously that freeze dyring can be done without destroying or creating radicals. Should this be accomplished it may be possible to use lyophilisates to expose tissue radicals to spin traps.

We would like to thank Prof. W. Lohmann for permitting to use the facilities of his institute, and Prof. H.W. Pia for allowing access to excised tissue. We are also grateful for the help received from Prof. W. Weis, Dr H. Neubacher, the Drs M. Mirbach and A. Schmidl (surface area), the many surgeons, and technical assistants.

**References**

BAYSAL, B.M., ERSON, K., KUCUKYAVUZ, S. & BAYSAL, M. (1979). ESR spectra of whole blood from normal and tumorous patients. *METU J. Pure Appl. Sci.*, **12**, 1.

BHAKOO, K.K., CROCKARD, H.A. & LASCELLES, P.T. (1984). Regional studies of changes in brain fatty acids following experimental ischemia and reperfusion in the gerbil. *J. Neurochem.*, **43**, 1025.

BLUMENFELD, L.A. (1981). *Problems of Biological Physics*, p. 126. Springer: Berlin, Heidelberg.

BRAU, R.H., GARCIA-CASTINEIRAS, S. & RIFKINSON, N. (1984). CSF ascorbic acid levels in neurological disorders. *Neurosurgery*, **14**, 142.

BRUNAUER, S., EMMETT, P. & TELLER, E. (1938). Adsorption of gases in multimolecular layers. *J. Am. Chem. Soc.*, **60**, 309.

CHAN, P.H., SCHMIDLEY, J.W., FISHMAN, R.A. & LONGAR, S.M. (1984). Brain injury, edema, and vascular permeability changes induced by oxygen derived free radicals. *Neurology*, **34**, 315.

CHETVERIKOV, A.G. (1964). Study of the spectra of EPR of biological specimens. *Biofizika*, **9**, 678. (engl. p. 738).

CIMBOLAITYTE, J.J., NAKTINIS, J.J., CERNIAUSKIENE, L.C. & VANIN, A.F. (1982). Nature of free radicals detected in dried animal tissues. *Biofizika*, **27**, 800. (engl. p. 839).

DODD, N.J.F. & SWARTZ, H.M. (1984). The nature of the ESR signal in lyophilized tissue and its relevance to malignancy. *Br. J. Cancer*, **49**, 65.

VON FOERSTER, G., WEIS, W. & STAUDINGER, H. (1966). Kinetik der Entstehung von Semidehydro-ascrobinsaeure. *Hoppe-Seylers Z. Physiol. Chem.*, **344**, 217.

GREULICH, W. (1980). *ESR und Atomabsorptions-messungen von Vitamin C und Erythrocytenmembranen*. Diplomarbeit, Department of Biophysics, Justus-Liebig-University, Giessen.

HALLIWELL, B. (1984). Oxygen radicals: A common sense look at their nature and medical importance. *Med. Biol.*, **62**, 71.

HECKLY, R.J. (1976). Free redicals in dry biological systems. In *Free Radicals in Biology*, Pryor, W.A. (ed) **2**, p. 135. Academic: New York.

KALUS, W.H. & FILBY, W.G. (1981). Is there an equilibrium between ascorbic and dehydroascorbic acids? *Z. Naturforsch. (C)*, **36**, 1088.

KAWAKATSU, M., TERAO, J. & MATSUSHITA, S. (1984). Phospholipid oxidation catalyzed by ferrous ion and ascorbic acid. *Agric. Biol. Chem.*, **48**, 1275.

KLATZO, I., PIRAUX, A. & LASKOWSKI, E.J. (1958). The relationship between edema, blood brain barrier, and tissue elements in a local brain injury. *J. Neuropath. Exp. Neurol.*, **17**, 548.

KUBO, K., YOSHITAKE, I., KUMADA, Y., SHUTO, K. & NAKAMIZO, N. (1984). Radical scavenging action of flunarizine in rat brain in vivo. *Arch. Int. Pharmacodyn.*, **272**, 283.

LOHMANN, W. (1976). Leukemia and the reducing properties of viruses. *Radiat. Environ. Biophys.*, **13**, 281.

LOHMANN, W. (1984). Structure of ascorbic acid and its biological function VI. Its importance for the $Na^+/K^+$-transport. *Biophys. Struct. Mech.*, **10**, 205.

NEUBACHER, H. (1984). ESR investigations on lyophilized blood: Mixtures with ascorbic acid. *Z. Naturforsch. (C)*, **39**, 174.

RUUGE, E.K. & BLYUMENFEL'D, L.A. (1965). Free radicals of ascorbic acid appearing on interaction with protein. *Biofizika*, **10**, 689.

SAMUNI, A., ARONOVITCH, J., GODINGER, D., CHEVION, M. & CZAPSKI, G. (1983). On the cytotoxicity of vitamin C and metal ions, a site specific Fenton mechanism. *Eur. J. Biochem.*, **137**, 119.

SASAKI, R., KUROKAWA, T. & TEROKUBOTA, S. (1982). Nature of serum ascorbate radical and its quantitative estimation. *Tohoku J. Exp. Med.*, **136**, 113.

SULLIVAN, S.G. & STERN, A. (1982). Effects of ascorbate on methemoglobin reduction in intact red cells. *Arch. Biochem. Biophys.*, **213**, 590.